# Probabilistic Fatigue Life Updating for Railway Bridges Based on Local Inspection and Repair

**DOI:** 10.3390/s17040936

**Published:** 2017-04-24

**Authors:** Young-Joo Lee, Robin E. Kim, Wonho Suh, Kiwon Park

**Affiliations:** 1 School of Urban and Environmental Engineering, Ulsan National Institute of Science and Technology (UNIST), Ulsan 44919, Korea; ylee@unist.ac.kr; 2 Fire Research Institute, Korea Institute of Civil Engineering and Building Technology, Hwaseong 18544, Korea; robineunjukim@kict.re.kr; 3 Department of Transportation and Logistics Engineering, Hanyang University ERICA Campus, Ansan 15588, Korea; 4 Department of Mechanical and Aerospace Engineering, Trine University, Angola, IN 46703, USA; parkk@trine.edu

**Keywords:** railway bridge, fatigue life updating, inspection and repair, system reliability

## Abstract

Railway bridges are exposed to repeated train loads, which may cause fatigue failure. As critical links in a transportation network, railway bridges are expected to survive for a target period of time, but sometimes they fail earlier than expected. To guarantee the target bridge life, bridge maintenance activities such as local inspection and repair should be undertaken properly. However, this is a challenging task because there are various sources of uncertainty associated with aging bridges, train loads, environmental conditions, and maintenance work. Therefore, to perform optimal risk-based maintenance of railway bridges, it is essential to estimate the probabilistic fatigue life of a railway bridge and update the life information based on the results of local inspections and repair. Recently, a system reliability approach was proposed to evaluate the fatigue failure risk of structural systems and update the prior risk information in various inspection scenarios. However, this approach can handle only a constant-amplitude load and has limitations in considering a cyclic load with varying amplitude levels, which is the major loading pattern generated by train traffic. In addition, it is not feasible to update the prior risk information after bridges are repaired. In this research, the system reliability approach is further developed so that it can handle a varying-amplitude load and update the system-level risk of fatigue failure for railway bridges after inspection and repair. The proposed method is applied to a numerical example of an in-service railway bridge, and the effects of inspection and repair on the probabilistic fatigue life are discussed.

## 1. Introduction

Railway bridges, which play an important role in the daily lives of numerous people, are known to be prone to the risk of fatigue failure. Many structural systems are subjected to the risk of fatigue-induced failure caused by repeated loading over their life cycle, which is a particularly critical safety concern for railway bridges. Several methods have been proposed for the fatigue damage mitigation of structures such as vibration control (e.g., tuned mass dampers) [1], local stress reduction (e.g., crack-arrest holes) [2], and compressive stress introduction (e.g., peening) [3]. To make appropriate decisions about effective bridge maintenance and repair, it is required to predict the fatigue life accurately. However, fatigue life prediction is a challenging task because such a prediction for a bridge requires considering results of inspection and repair, which are affected by various sources of uncertainty, including material properties, anticipated vehicle loads, and environmental conditions.

Two approaches are commonly employed for the fatigue damage evaluation and life prediction of bridge structures. The first approach is the traditional stress–life (S–N) curve method, in which the relationship between the constant-amplitude stress range, *S*, and the number of cycles to failure, *N*, is determined by performing appropriate fatigue experiments and described by an S–N curve. In this approach, the Palmgren–Miner linear damage hypothesis, also called Miner’s rule [4], extends this approach to varying-amplitude loadings. The second method is the fracture mechanics approach, which is dominantly dedicated to exploring the features and disciplines of crack initiation and growth while considering the stress field at the crack tip. 

In general, the two approaches are applied sequentially, with the S–N curve method being used at the bridge design stage or for the preliminary evaluation of fatigue life and the fracture mechanics approach being used for more refined crack-based assessment of the remaining fatigue life or for effective decision-making on inspection and maintenance strategies [5]. The S–N curve is obtained entirely from experiments and has limitations in estimating the probabilistic bridge life based on the current condition of a bridge. Meanwhile, the fracture mechanics approach is usually applied to predict the propagation life from an initial crack or defect. The method based on linear elastic fracture mechanics (LEFM) relates the growth of an initial crack of size to the number of fatigue cycles. Many investigations have been performed on bridge fatigue condition assessment using the fracture mechanics approach. Fisher [6] illustrated more than 25 case studies on fatigue crack phenomena in steel bridges using the fracture mechanics approach and other theories. Agerskov and Nielsen [7] investigated the fatigue damage accumulation in steel bridges under random loadings and determined the fatigue life of welded joints in steel highway bridges by carrying out fracture mechanics analysis. By using an LEFM model to predict crack growth, MacDougall et al. [8] quantified the differences between the fatigue lives of a short-span bridge and a medium-span bridge under successive passages of either a steel-sprung vehicle or an air-sprung vehicle. Using experimental data obtained from the structural components on the Kinuura Bridge and theoretical predictions based on LEFM, Xiao et al. [9] found that the lack of penetration zones of 2–3 mm resulted in the low fatigue strength of butt-welded joints.

Structural health monitoring (SHM) systems have often been used to probabilistically evaluate the fatigue life of a bridge based on the measurement of its current condition [10]. LEFM approaches are more suitable for fatigue life evaluation than S–N curve approaches because LEFM can describe fatigue crack propagation quantitatively in terms of the crack length, whereas the S–N curve can only identify whether the structure will fail or survive. Recently, long-term SHM of bridges has been one of the major topics of interest for researchers and engineers in the fields of civil, mechanical, materials, and computer science engineering [11]. The design and implementation of such an SHM system involve the integration of analytical skills and instrumentation technologies with the knowledge and experience of bridge design, construction, inspection, maintenance, and management. An online SHM system can provide reliable information pertaining to the integrity, durability, and reliability of bridges. The information can then be incorporated into a bridge management and maintenance system for optimizing maintenance actions and improving design standards, specifications, codes, and guidelines. The SHM system is, in fact, an augmentation but not a substitute for systems used in current practice in bridge maintenance and management, not only through the use of advanced technologies in sensing, data acquisition, computing, communication, and data and information management but also through the effective integration of these technologies into an intelligent system. In addition, online SHM strategies have been applied to detect structural damage by system identification and model updating [12,13,14]. An accurate estimation of the actual situation pertaining to the fatigue life and remaining life of critical components is an important task of the SHM system; this task can be accomplished by using the continuously measured data of dynamic strain from the long-term SHM system. The fatigue performance of steel bridges depends on a number of factors such as material characteristics, stress history, and environmental conditions, and all these factors exhibit uncertainty and randomness during the service life of the bridge. On the other hand, when field measurement data are used for fatigue condition assessment, the uncertainties related to the data and the inaccuracies resulting from the use of data-processing techniques persist and are hardly avoidable. In view of these facts, it is more appropriate to conduct fatigue life assessment in a probabilistic manner rather than in a deterministic manner.

However, the results of bridge inspection and repair, which is one of the important SHM datum, are not used in the abovementioned studies. Lee and Song [15] proposed a new system reliability approach for updating the structural fatigue life by using formulations for fatigue life evaluation. In their research, however, the proposed method was applied to a structure where the loading condition was simplified as a constant amplitude. In addition, it is not feasible to update the prior risk information after bridges are repaired.

In this paper, a new method is proposed for the effective fatigue life evaluation of railway bridges by addressing the following challenges: (i) the formulations proposed by Lee and Song [15] are further developed so that the proposed method can handle varying-amplitude loads; and (ii) the proposed method enables updating the system-level risk of fatigue failure for railway bridges after inspection and repair. To demonstrate the method, it is applied to a numerical example of an in-service railway bridge. SHM data for the railway bridge subjected to actual train loading are introduced and analyzed by using rainflow counting, and the fatigue life of the bridge is evaluated and updated based on various results of inspection and repair.

## 2. Proposed Methodology

### 2.1. Limit-State Function Formulations for Fatigue Failure

In a structural reliability problem, the failure probability calculation requires constructing the so-called limit-state function, which represents the failure event of interest as an analytical function of random variables and deterministic parameters [10,15]. As mentioned in Section 1, many methods for the fatigue reliability analysis of bridges have been developed based on the S–N or LEFM approach. A widely used LEFM-based numerical model for crack propagation is the Paris equation [16]. Based on this equation, Lee and Cho [10] and Lee and Song [15] derived a series of formulations for estimating the structural risk of fatigue-induced failure. However, the formulations are based on constant-amplitude loads, and they cannot be applied to a railway bridge because they cannot consider cyclic loads with varying amplitudes, which is a realistic pattern generated by train traffic. Therefore, in this study, the limit-state functions proposed by Lee and Cho [10] and Lee and Song [15] are further developed for evaluating the fatigue life of a railway bridge with varying-amplitude loads.

First, consider the following Paris equation [16], which is a widely-known model of crack propagation:

(1) $$\frac{da}{dN}=C{\left(\Delta K\right)}^{m}$$

where *a* denotes the crack length, *N* denotes the number of loading cycles, *C* and *m* represent the material properties, and Δ*K* is the range of the stress intensity factor. This range can be evaluated by using Newman’s approximation [17]:

(2) $$\Delta K=S\cdot Y(a)\cdot \sqrt{\pi a}$$

where Δ*S* denotes the stress amplitude and *Y*(*a*) is the geometry function, which is a dimensionless parameter that depends on the geometry around the crack. The following equation can be obtained by substituting Equation (2) into Equation (1):

(3) $$\frac{1}{{\left[Y(a)\sqrt{\pi a}\right]}^{m}}da=C\cdot \Delta S^{m}dN$$

**Figure 1:**
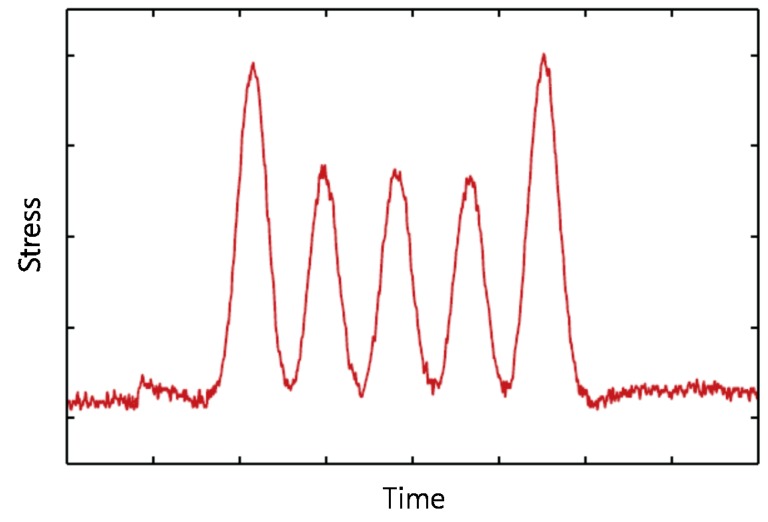
Generic stress response of a railway bridge under passing train.

If the load amplitude is a constant, Equation (3) can be developed to an equation for the fatigue life evaluation of a structure, as described by Lee and Song [15]. However, when a train passes over a bridge, the stress experienced by a structural member is found to be fluctuating, as shown in Figure 1, and this makes it impossible to use the formulations devised for a constant amplitude.

The rainflow cycle counting algorithm [18] is widely used to handle such a varying-amplitude load and assess the fatigue life of structures, especially in mechanical engineering. Usually, the algorithm extracts cycles from the load, stress, or strain history obtained from measurement or simulation results. From the cycle counting, several cycles and half-cycles with different amplitude and mean values are obtained. This counting algorithm can be used with the Paris equation, which enables it to compute the expected fatigue life under random loading conditions in a theoretical manner [18].

The stress amplitude Δ*S_i_* and the corresponding number of cycles *n_i_* are used for the integration of Equation (3) from the initial condition to the current time point, thereby providing the following relationship between the current crack length and the time duration as follows:

(4) $$\int_{a^{0}}^{a}\frac{1}{{\left[Y(a)\sqrt{\pi a}\right]}^{m}}da=C\cdot \nu_{0}\cdot T\cdot \sum_{i=1}^{N_{amp}}\left[n_{i}{\left(\Delta S_{i}\right)}^{m}\right]$$

where *a*^0^ is the initial crack length, ν_0_ is the frequency of train loading, *T* is the time duration, and *N_amp_* is the total number of stress amplitude values. If a crack failure is assumed to occur when the crack length exceeds the critical crack length *a^c^*, the time required for crack growth from *a*^0^ to *a^c^*, *T*, is expressed by:

(5) $$T=\frac{1}{C\nu_{0}\sum_{i=1}^{N_{amp}}\left[n_{i}{\left(\Delta S_{i}\right)}^{m}\right]}\int_{a^{0}}^{a^{c}}\frac{1}{{\left[Y(a)\sqrt{\pi a}\right]}^{m}}da$$

The limit-state function for the failure of the *j*-th structural member or location (“member” is used hereafter) within a given time interval [0, *T_s_*] is expressed by:

(6) $$g(X)=T_{j}^{0}-T_{s}=\frac{1}{C\nu_{0}\sum_{i=1}^{N_{amp}}\left[n_{i}{\left(\Delta S_{i}\right)}^{m}\right]}\int_{a^{0}}^{a^{c}}\frac{1}{{\left[Y(a)\sqrt{\pi a}\right]}^{m}}da-T_{s}$$

where **X** denotes the vector of random variables and *T_j_*^0^ is the time required for the fatigue failure of the *j*-th structural member. In structural reliability problems, *g*(**X**) ≤ 0 typically indicates the occurrence of a failure event.

Although several studies [10,15,19,20] for the probability estimation of fatigue failure events were conducted with the Paris equation in Equation (1), other crack propagation models such as the Walker equation [21] and the Forman equation [22] can also be introduced to model crack growth more accurately. Various crack growth models have been suggested to improve the Paris equation, and any model can replace the Paris equation in the proposed method, if it satisfies the following conditions: (1) the time duration of the crack growth can be analytically expressed in terms of random variables and deterministic variables, as done in Equations (1)–(5); and (2) the statistical properties (e.g., distribution type, mean, standard deviation, and correlation) of the random variables are available. The first condition can be met by several crack growth models. In this research, however, the Paris equation is selected because the statistical characteristics of the equation parameters were investigated sufficiently as discussed in the previous studies [10,15].

If a railway bridge is considered to be a structural system consisting of multiple structural members, the failure of the bridge system should be expressed by a Boolean function of the failure events of individual members; to obtain such a function, system reliability analysis is required. In this research, a bridge is considered to fail if any of its members fails because of fatigue, and the probability of this system event, *P_sys_*, is described as:

(7) $$P_{sys}=P\left[\overset{N_{mem}}{\underset{j=1}{\cup }}\left(T_{j}^{0}<T_{s}\right)\right]$$

where *N_mem_* is the total number of members in consideration.

### 2.2. Reliability Updating through Inspection and Repair Events

A new approach to update the probability of fatigue failure based on inspection results was recently proposed [15]. However, the formulations provided in the research were developed for aircraft and cannot be used to update the prior risk information after repairing events. In the present research, the previously proposed formulations are introduced and then further developed so that it can be applied to railway bridges after both of inspection and repairing events.

For an event of interest *E_i_*, (e.g., a member failure in Equation (6) or a system failure in Equation (7)), its probability *P_i_* can be updated through an inspection event as follows:

(8) $$P_{i,up}=P\left(E_{i}|IE_{j}\right)$$

where *IE_j_* denotes the inspection event for the *j*-th member. This formulation can be extended to utilize multiple available inspection results to update the probability *P_i_*, i.e.,:

(9) $$P_{i,up}=P\left(E_{i}|IE_{j}\cap IE_{k}\cap \cdot \cdot \cdot \cap IE_{l}\right)$$

Inspection results are classified into two types of events—*equality* and *inequality* events—depending on whether a crack is detected (and measured) or not. Considering that inspections are generally made at multiple locations, three possible combinations of inspection results exist: *inequality*, *equality*, and *mixed* cases [15,23].

First, an inequality event indicates that no crack is detected through inspection at a certain single location. There are two possible explanations as to why no crack is detected. First, a crack is too small to be detected. Second, although a relatively large crack actually exists on the inspected member, it may be missed because of human error or the limitations of the detecting device. In either case, this event can be formulated as:

(10) $$\begin{array}{l} IE_{j}:g_{j,no}\left(X\right)=T_{I}-T_{j}^{d}\left(X\right)<0\;\\ \mathrm{where}\;\;T_{j}^{d}=\frac{1}{C\nu_{0}\sum_{i=1}^{N_{amp}}\left[n_{i}{\left(\Delta S_{i}\right)}^{m}\right]}\int_{a_{j}^{0}}^{a^{d}}\frac{1}{{\left[Y(a)\sqrt{\pi a}\right]}^{m}}da \end{array}$$

where *T_j_^d^* denotes the time required for the crack to grow to a detectable crack size in the *j*-th member, *a^d^* is the detectable crack size during the inspection process, and *T_I_* denotes the time at which the member is inspected. The detectable crack size *a^d^* is related to a specific inspection method and is modeled as a random variable reflecting the actual probability of detection.

For a single inequality event, the conditional probability in Equation (8) can be calculated as:

(11) $$P_{i,up}=P\left(E_{i}|IE_{j}\right)=\frac{P\left(E_{i}\cap \left(g_{j,no}\left(X\right)<0\right)\right)}{P\left(g_{j,no}\left(X\right)<0\right)}$$

Likewise, the conditional probability for multiple inequality events can be calculated as:

(12) $$\begin{array}{l} P_{i,up}=P\left(E_{i}|IE_{j}\cap \cdot \cdot \cdot \cap IE_{k}\right)\\ \;\;\;\;\;\;\;=\frac{P\left[E_{i}\cap \left(g_{j,no}\left(X\right)<0\right)\cap \cdot \cdot \cdot \cap \left(g_{k,no}\left(X\right)<0\right)\right]}{P\left[\left(g_{j,no}\left(X\right)<0\right)\cap \cdot \cdot \cdot \cap \left(g_{k,no}\left(X\right)<0\right)\right]} \end{array}$$

The updated probabilities can be obtained from Equations (11) and (12) by computing the probabilities in the numerator and the denominator by component and system reliability analysis.

Second, an equality event indicates that a crack is detected and measured. The event is formulated as:

(13) $$\begin{array}{l} IE_{j}:g_{j,yes}\left(X\right)=T_{I}-T_{j}^{m}\left(X\right)=0\\ \mathrm{where}\;\;T_{j}^{m}=\frac{1}{C\nu_{0}\sum_{i=1}^{N_{amp}}\left[n_{i}{\left(\Delta S_{i}\right)}^{m}\right]}\int_{a_{j}^{0}}^{a^{m}+\varepsilon^{m}}\frac{1}{{\left[Y(a)\sqrt{\pi a}\right]}^{m}}da=0 \end{array}$$

where *T_j_^m^* denotes the time required for the crack to grow to the measured crack size in the *j*-th member, *a^m^* is the measured crack size, and ε*^m^* is the measuring error. In the equation, it should be noted that *T_j_^m^* is equal to the inspection time *T_I_*, which makes the formulation different from the limit-state formulations in Equation (10). The conditional probability for the equality event is calculated as:

(14) $$P_{i,up}=P\left(E_{i}|IE_{j}\right)=\frac{P\left(E_{i}\cap \left(g_{j,yes}\left(X\right)=0\right)\right)}{P\left(g_{j,yes}\left(X\right)=0\right)}$$

Compared to the terms in Equation (11), the probability terms in Equation (14) are more challenging to compute using structural reliability methods because the probabilities of both the numerator and the denominator are zero [24]. Through an analytical derivation presented in a previous study [15], Equation (14) is transformed to:

(15) $$P_{i,up}={\left[\frac{\frac{\partial }{\partial \theta }P\left[E_{i}\cap \left(g_{j,yes}+\theta \leq 0\right)\right]}{\frac{\partial }{\partial \theta }P\left[\left(g_{j,yes}+\theta \leq 0\right)\right]}\right]}_{\theta =0}$$

The updated probability in the equation can be calculated via numerical differentiation. Likewise, the conditional probability for multiple equality events is formulated as follows [25]:

(16) $$\begin{array}{l} P_{i,up}=P\left(E_{i}|IE_{j}\cap \cdot \cdot \cdot IE_{k}\right)\\ \;={\left[\frac{\frac{\partial^{n}}{\partial \theta_{1}\cdot \cdot \cdot \partial \theta_{n}}P\left[E_{i}\cap \left(g_{j,yes}+\theta_{1}\leq 0\right)\cap \cdot \cdot \cdot \cap \left(g_{k,yes}+\theta_{n}\leq 0\right)\right]}{\frac{\partial^{n}}{\partial \theta_{1}\cdot \cdot \cdot \partial \theta_{n}}P\left[\left(g_{j,yes}+\theta_{1}\leq 0\right)\cap \cdot \cdot \cdot \cap \left(g_{k,yes}+\theta_{n}\leq 0\right)\right]}\right]}_{\theta_{1}=\cdot \cdot \cdot \theta_{n}=0} \end{array}$$

where *n* is the number of observed equality events. The updated probability *P_i,up_* in Equation (16) can also be calculated using the *n*-th-order numerical differentiation. However, the equation needs to be used with caution because such a high-order numerical differentiation can produce significant error unless the probability calculations in the numerator and the denominator are extremely accurate. Therefore, the equality case including multiple equality events is not discussed in this research.

Lastly, with regard to mixed cases, the simplest case involves a single inequality event and a single equality event. The updated probability is formulated as:

(17) $$P_{i,up}=\frac{P\left[E_{i}\cap \left(g_{j,no}\left(X\right)<0\right)\cap \left(g_{j,yes}\left(X\right)=0\right)\right]}{P\left[\left(g_{j,no}\left(X\right)<0\right)\cap \left(g_{j,yes}\left(X\right)=0\right)\right]}$$

This equation can be transformed to:

(18) $$P_{i,up}={\left[\frac{\frac{\partial }{\partial \theta }P\left[E_{i}\cap \left(g_{j,no}<0\right)\cap \left(g_{j,yes}+\theta \leq 0\right)\right]}{\frac{\partial }{\partial \theta }P\left[\left(g_{j,no}<0\right)\cap \left(g_{j,yes}+\theta \leq 0\right)\right]}\right]}_{\theta =0}$$

Finally, this formulation can be generalized for a mixed case involving multiple inequality and equality inspection events as:

(19) $$\begin{array}{l} P_{i,up}=\{\frac{\partial^{n}}{\partial \theta_{1}\cdot \cdot \cdot \partial \theta_{n}}P[E_{i}\cap \left(g_{j,no}<0\right)\cap \cdot \cdot \cdot \cap \left(g_{k,no}<0\right)\cap \\ \;\;\;\;\;\;\;\;\;\;\;\;\;\;\;\;\;\;\;\;\;\;\left(g_{l,yes}+\theta_{1}\leq 0\right)\;\cap \cdot \cdot \cdot \cap \;\left(g_{l,yes}+\theta_{n}\leq 0\right)]/\\ \;\;\;\;\;\;\;\;\;\;\frac{\partial^{n}}{\partial \theta_{1}\cdot \cdot \cdot \partial \theta_{n}}P[\left(g_{j,no}<0\right)\cap \cdot \cdot \cdot \cap \left(g_{k,no}<0\right)\cap \\ \;\;\;\;\;\;\;\;\;\;\;\;\;\;\;\;\;\;\;\;\;\;{\left(g_{l,yes}+\theta_{1}\leq 0\right)\;\cap \cdot \cdot \cdot \cap \;\left(g_{l,yes}+\theta_{n}\leq 0\right)\}}_{\theta_{1}=\cdot \cdot \cdot \theta_{n}=0} \end{array}$$

It should be noted that Equation (19) is the combination of Equations (12) and (16). Because of the aforementioned difficulty in carrying out high-order numerical differentiation, the mixed case involving multiple equality events is not handled in this research.

Overall, system reliability updating after repair is similar to that through inspection. However, when a crack is detected, measured, and repaired, the crack size in the repaired member changes; therefore, structural analysis needs to be performed with a new initial crack length for the repaired member. When the repair is made at *T_r_*, according to Equation (6), the limit-state function for the failure of the repaired member *j* within an inspection cycle [0, Ts] is described as:

(20) $$\begin{array}{l} g\left(X\right)=T_{j}^{r}+T_{r}-T_{s}\;\\ T_{j}^{r}=\frac{1}{C\nu_{0}\sum_{i=1}^{N_{amp}}\left[n_{i}{\left(\Delta S_{i}\right)}^{m}\right]}\int_{a_{j}^{r}}^{a_{j}^{c}}\frac{1}{{\left[Y(a)\sqrt{\pi a}\right]}^{m}}da \end{array}$$

where *a_j_^r^* is the initial crack length in the repaired member. *a_j_^r^* depends on the quality of the repair methods, e.g., grinding and welding.

In summary, the fatigue failure events of a structural member and structural system are expressed by analytical functions of random variables and deterministic parameters shown in Equations (6) and (7), respectively. These equations are derived through the analytical derivation from Equation (1). Likewise, the events of no-crack detection, crack detection and size measurement, and repair are expressed by Equations (10), (13) and (20), respectively. With these equations, the original probability of a fatigue failure event which can be estimated from Equations (6) and (7) can be updated based on the conditional probability, as shown in Equations (9), (16) and (19).

A reliability analysis needs to be performed to calculate the probability of a fatigue failure event with these formulations, and numerous reliability analysis methods have been developed so far [26]. Among them, the first-order reliability method (FORM) [27] and the second-order reliability method (SORM) [28] are used to perform component reliability analysis. In this research, FERUM (Finite Element Reliability Using MATLAB) [29], which is a reliability software package developed at the University of California, Berkeley, was modified and then introduced to perform reliability analysis. In addition, for a structural system consisting of multiple structural members, a failure event needs to be expressed by a system event which requires system reliability analysis (SRA) [30,31]. In this study, it is assumed that the railway bridge system fails if any of its structural members fail, and the Matrix-based System Reliability (MSR) method [31] is employed to calculate the system probability.

## 3. Numerical Example

### 3.1. Example of Bridge Considered in This Study: Calumet Bridge

**Figure 2:**
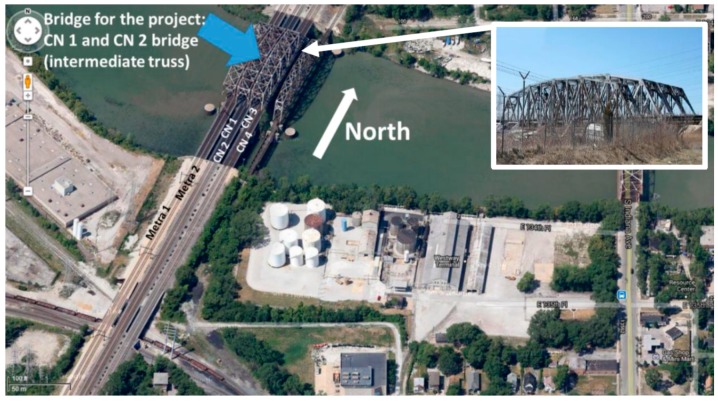
Test bridge (i.e., Calumet Bridge) marked as CN1 and CN2 [32,33].

The proposed method of fatigue life updating is tested by applying it to a real bridge named Calumet Bridge located in Illinois, USA. The bridge is located over the Little Calumet River near Chicago, IL (Figure 2). 

The following four bridges are shown in Figure 2: the test bridge is the second to left truss bridge (the bridge marked with an arrow), the west-most bridge is used for the Metra lines, another truss bridge carrying freight and passenger trains is located to the east of the test bridge, and the east-most bridge is closed for traffic. In Kim et al. and Moreu et al. [32,33], the structural condition of the Calumet Bridge was assessed based on in-service data obtained from an SHM system.

The bridge is approximately 95 m long, 21 m tall, and 10 m wide and is made of A36 American Society for Testing and Materials (ASTM) steel. The bridge opened for service in 1971, with an expected life of 100 years. The test bridge carries two tracks—the CN 1 track on the west side and the CN 2 track on the east side—both of which are open for south- and north-bound freight and passenger trains. Approximately ten freight trains and six passenger (Amtrak) trains run on either the CN 1 track or the CN 2 track on a daily basis. The following aspects of the bridge make it suitable and unique for use as the test bridge: (i) The test bridge is made of steel, which is the most common type of bridge construction material used in the U.S., but vulnerable to fatigue; (ii) Only the train loads are applied to the test bridge, whereas adjacent bridges are open to car traffic as well; (iii) A large amount of traffic comprising various types of trains on the daily basis allows rich types of excitation on the bridge. This bridge handles a large amount of traffic per day (approximately 20 trains, including both freight and Amtrak traffic).

Wireless smart sensor (WSS) networks were installed for a year on the bridge for creating an efficient SHM system with various types of sensors [32]. The wireless Imote2 platform was used in the research [34]. This platform can stack a sensor board via two external connectors, thus allowing the network to have a flexible design with any type of sensor board. The sensor boards used were (i) a normal accelerometer board; (ii) a high-sensitivity accelerometer board; and (iii) a strain sensor (denoted as SHM-S sensor).

Using the measured data from the WSS networks, the authors in Kim et al. [35] developed and calibrated a model that can efficiently estimate structural behavior subject to a moving train. The model was validated by comparing the measured axial strain from the network with the estimated strain from the model. The model is adopted in this paper, and the maximum axial strains estimated from the model, while a test car in Kim et al. [32] ran at 53.11 km/h is used in the paper. More details regarding the bridge including section properties and the model can be found in [32,35].

### 3.2. Monitoring Data and Rainflow Counting

**Figure 3:**
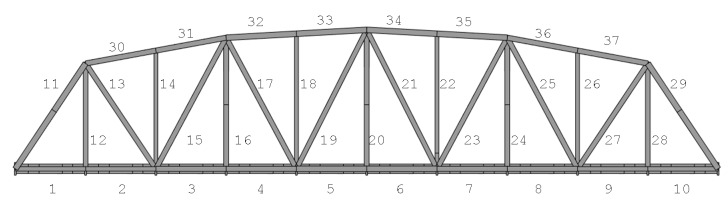
Locations of 10 members with the maximum stresses.

**Figure 4:**
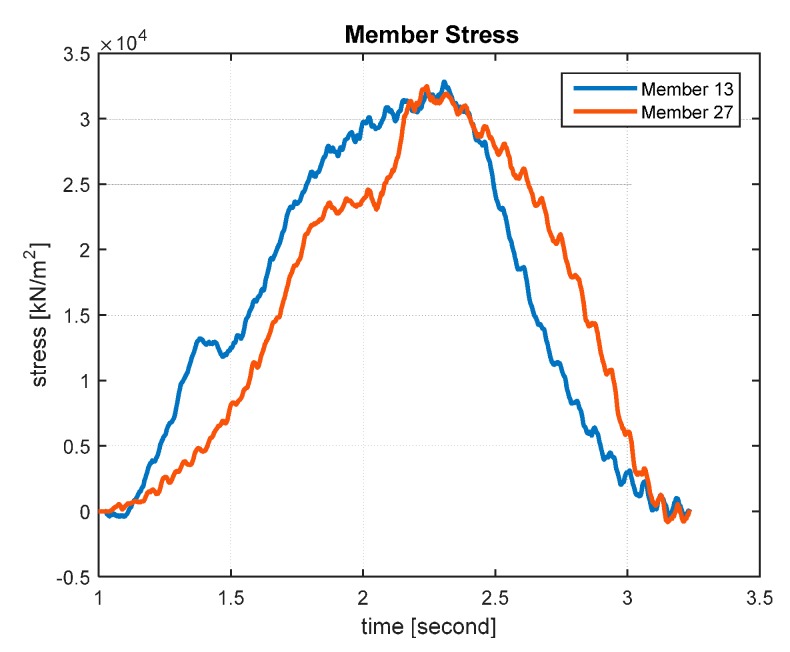
Stress history of Members 13 and 27.

Calumet Bridge consists of over hundreds of members, and it is not feasible to monitor all the members. Therefore, in this research, 10 global members in the calibrated model that show the highest levels of axial stresses under a test train [32] are selected for monitoring. The 10 members in the decreasing order of their maximum stresses are Members 13, 27, 8, 6, 7, 5, 10, 9, 17 and 4, whose locations are shown in Figure 3. Table 1 lists the maximum stresses of the 10 members; the stress levels of Members 13 and 27 are much higher than those of the other members. In addition, Figure 4 shows the stresses of Members 13 and 27 under a passing train.

Next, the rainflow counting algorithm is applied to the stress responses of the 10 selected members. The rainflow cycle counting algorithm is widely used for the fatigue life assessment of machine components or structures under varying-amplitude loading. Usually, the algorithm extracts cycles from the load, stress, or strain history obtained from measurement or simulation. In this research, a code for rainflow counting developed by Nieslony [18] is used and applied to the simulated results using the model developed by Kim et al. [35], and the number of amplitudes and the number of cycles are then obtained. The data are used to calculate $\sum_{i=1}^{N_{amp}}\left[n_{i}{\left(\Delta S_{i}\right)}^{m}\right]$ in Equation (4).

### 3.3. Random Variables and Deterministic Parameters

In this research, the statistical information of random variables is determined through a comprehensive literature review [10,15,19,36,37,38], and the mean, coefficient of variation (c.o.v.), and distribution type of the random variables in this numerical example are summarized in Table 2. 

Although each of the random variables in this numerical example is assumed to follow the normal, lognormal, or exponential distribution, other distribution types such as Gumbel and Weibull distributions can also be introduced if they are more appropriate to represent the uncertainty of random variable. This is possible because the proposed method employs FERUM which enables the reliability with a total of 16 different probability distributions including normal, lognormal, exponential, Gumbel, and Weibull distributions.

In this example, only *C* is considered as a random variable and *m* is assumed to be a constant (3.344), because it was shown through a preliminary analysis that considering *m* as another random variable had a negligible effect on the failure probability result despite of a large computational cost.

Statistical independence is assumed among all the random variables, except the following variables: (1) values of the Paris equation parameter *C* of the 10 members (correlation coefficient: 0.6); (2) initial crack lengths (*a*^0^) in the 10 members (correlation coefficient: 0.6); (3) initial crack lengths in the repaired members (*a^r^*); (4) detectable crack sizes (*a^d^*); and (5) crack measurement errors (ε*^m^*). The correlation coefficients in these cases are not known, so they are initially assumed to be 0.6, which indicates that the 10 members are manufactured by the same manufacturer and that their material properties are thus highly correlated.

In addition, the following deterministic parameters are used: L-bracket width (*b*) = 650 mm, critical crack length (*a^c^*) = 12.7 or 19.05 mm, and average train traffic (assumed based on Moreu et al. [33]) = 20/day. For the geometry function *Y*(*a*) in Equation (2), the following function for I-beams is introduced based on Tada et al. [36]:

(21) $$Y\left(a\right)=1.122-0.231\left(\frac{a}{b}\right)+10.550{\left(\frac{a}{b}\right)}^{2}-21.710{\left(\frac{a}{b}\right)}^{3}+30.382{\left(\frac{a}{b}\right)}^{4}$$

## 4. Analysis Results

### 4.1. Fatigue Life Evaluation

Using the proposed method, the failure probabilities of the 10 selected members are calculated at various periods of the service time. Because the failure probabilities turned out to be very small, reliability index, a useful probability indicator which can be calculated by the following equation, is introduced:

(22) $$\beta =\Phi^{-1}\left(1-P_{f}\right)$$

where *β* is the reliability index, Φ^−1^(·) is the inverse function of the standard normal CDF (cumulative distribution function), and the *P_f_* is the failure probability.

**Figure 5:**
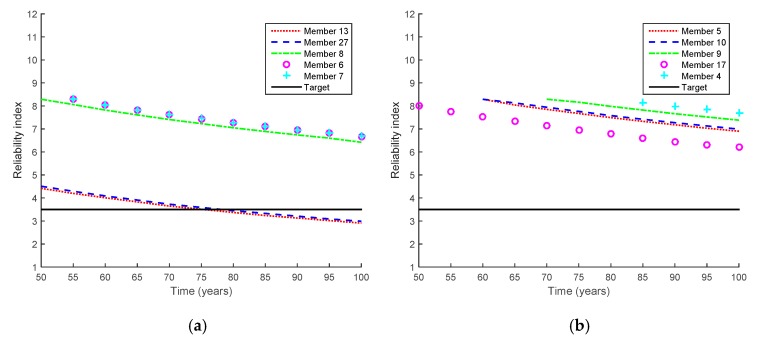
Reliability indices of 10 selected members obtained from proposed method. (**a**) Members 13, 27, 8, 6 and 7; (**b**) Members 5, 10, 9, 17 and 4.

Figure 5 shows the reliability indices of the 10 selected members at various periods of the service time. Overall, the reliability indices of the bridge decrease as the service life increases, which means that the probability of failure increases as the use of the bridge increases.

The American Association of State Highway and Transportation Officials (AASHTO) Bridge Design Code recommends a target reliability index of 3.5 (i.e., a failure probability of 2.33 × 10^−4^) with a service life of 75 years for steel and prestressed concrete components [10]. By finding the intersections between the reliability index curves and the target reliability index (i.e., black lines in Figure 5), the fatigue lives of all the 10 members are estimated. Accordingly, the fatigue lives of Members 13 and 27 are evaluated as 75.4 and 78.2 years, respectively, whereas the fatigue lives of the other members are estimated to be longer than 100 years. As mentioned in Section 3.2, this is mainly because the stress levels of Members 13 and 27 are much higher than those of the other members. Therefore, the analysis results of only Members 13 and 27 are presented hereafter.

**Figure 6:**
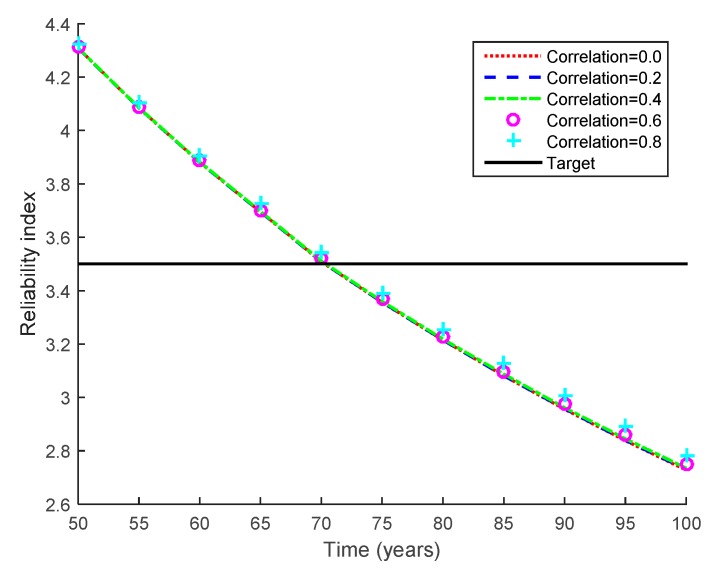
Reliability indices of bridge system with varying correlation coefficients.

In addition to the analysis results of the structural members, the fatigue life of the bridge system is evaluated. In the evaluation, as mentioned in Section 2.2, the system failure event is assumed to occur when any of the 10 members fails. Furthermore, the correlation coefficient between the values of the Paris equation parameter *C* and between the initial crack lengths *a*^0^ of the 10 members is assumed to be 0.6, which accounts for the high statistical dependence due to the assumption that all the members are produced by the same manufacturer. In addition, the fatigue life of the bridge system is evaluated using a range of correlation coefficient values (i.e., 0.0, 0.2, 0.4, 0.6 and 0.8). This evaluation is carried out to investigate the effect of the correlation coefficient as a parametric study, and the analysis results are shown in Figure 6.

The correlation coefficient has a negligible impact on the variations in the system bridge life against fatigue. This is because the system failure event is dominated by the failure events of Members 13 and 27, even though the failure event of the bridge system is assumed to occur if any of the 10 members fails. Therefore, in this example, the correlation coefficient is set to be 0.6 for the following analyses. With this correlation coefficient and the target reliability index of 3.5, the fatigue life of this bridge system is evaluated as 70.7 years.

### 4.2. Updated Fatigue Life Based on Local Inspection and Repair

To test the proposed method, it is applied to hypothetical scenarios of local inspection and repair, which are listed in Table 3.

#### 4.2.1. Inequality Cases

**Figure 7:**
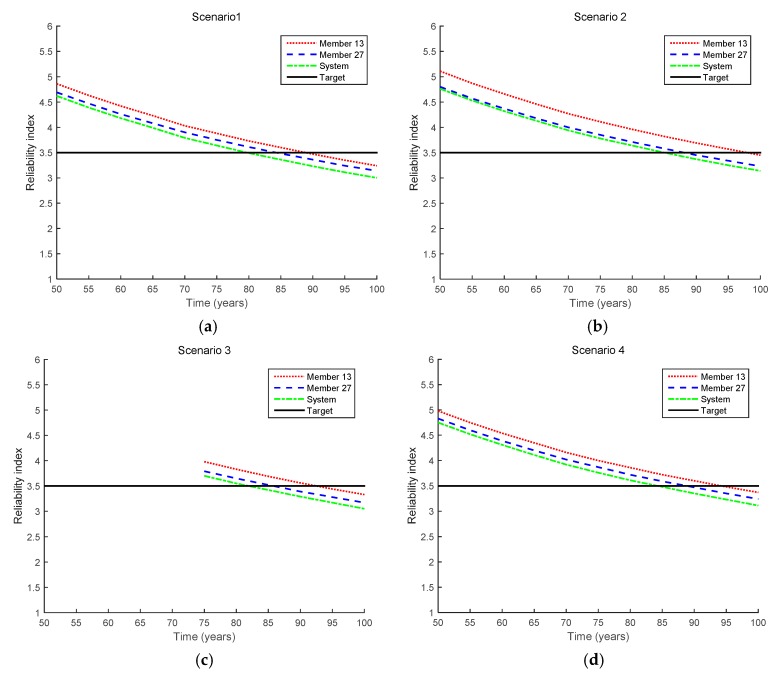
Reliability updating results by the proposed method for inequality cases (Scenarios 1–4 in Table 3). (**a**) Scenario 1; (**b**) Scenario 2; (**c**) Scenario 3; (**d**) Scenario 4.

Figure 7 shows the reliability index results of Members 13 and 27 and the bridge system for the inequality cases. First, the updated probabilities of different scenarios are compared with each other to investigate the impact of various inspection conditions on the reliability updating, such as the number of inspections, crack-detecting resolution, inspection interval, and measured crack length.

**Figure 8:**
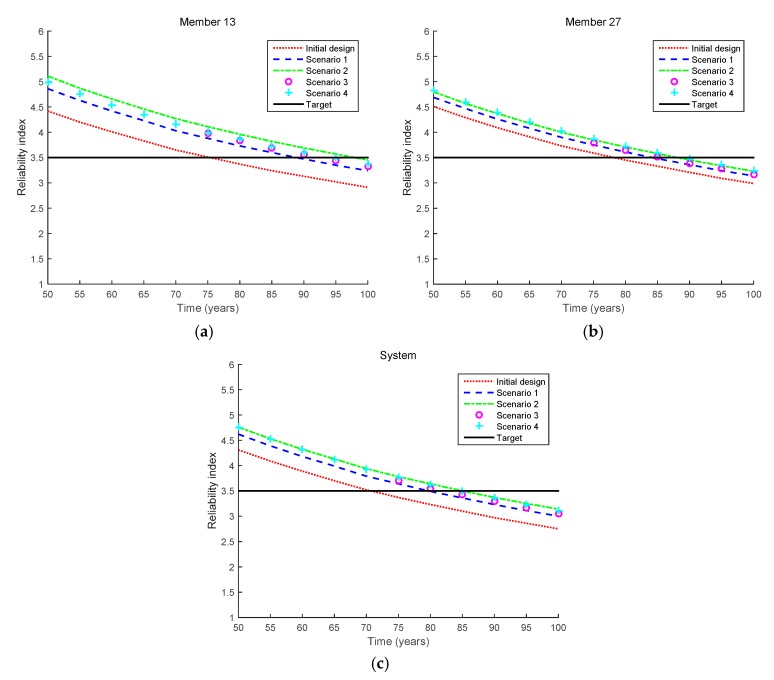
Comparison of updated reliability indices for inequality cases (Scenarios 1–4 in Table 3). (**a**) Member 13; (**b**) Member 27; (**c**) System.

To show the reliability index updates more clearly, Figure 8 shows how the reliability indices of Member 13, Member 27, and the bridge system are updated in Scenarios 1–4. In Scenario 1, the updated reliability indices decrease for the two members and the system because no crack is detected even after 50 years. The updated indices decrease further in Scenario 2 because no crack is detected even when a better crack-detecting device is used (with a smaller mean of the detectable crack size *a^d^*). In Scenario 3, no crack is detected even though an inspection is made at a later time (i.e., 75 years) than in Scenario 1, which further increases the reliability indices. In Scenario 4, it is assumed that a crack is not observed at any of the members. Because there are 10 members of interest, the inspection event is described as the intersection of 10 inequality events, as shown in Equation (12). Compared to Scenario 1, nine additional signs indicating the better safety of the bridge are obtained in Scenario 4, which further increases the reliability indices, as shown in Figure 8. From Figure 8, the fatigue lives of Member 13, Member 27, and the bridge system in Scenarios 1–4 can be evaluated, and the results are shown in Table 4.

#### 4.2.2. Equality Cases

**Figure 9:**
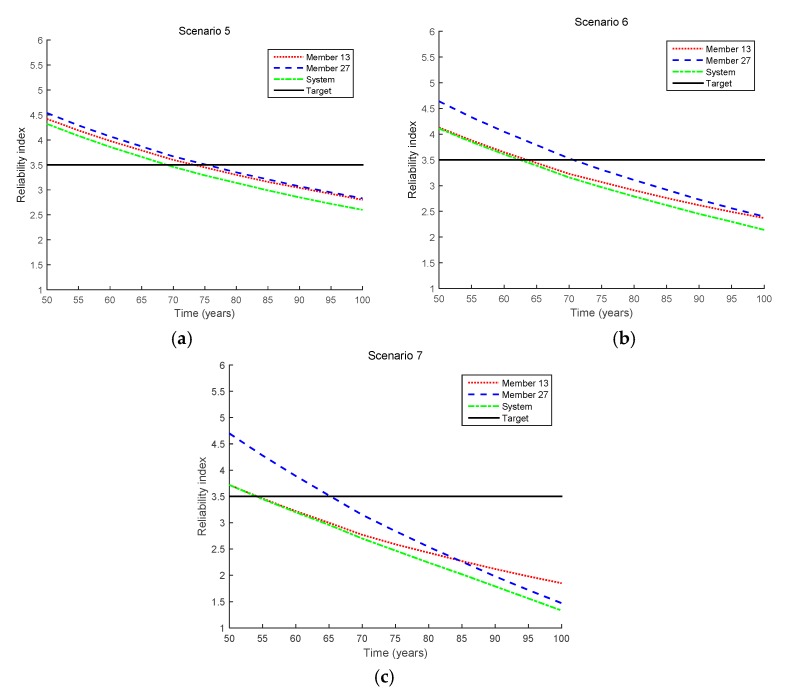
Reliability updating results by the proposed method for inequality cases (Scenarios 5–7 in Table 3). (**a**) Scenario 5; (**b**) Scenario 6; (**c**) Scenario 7.

**Figure 10:**
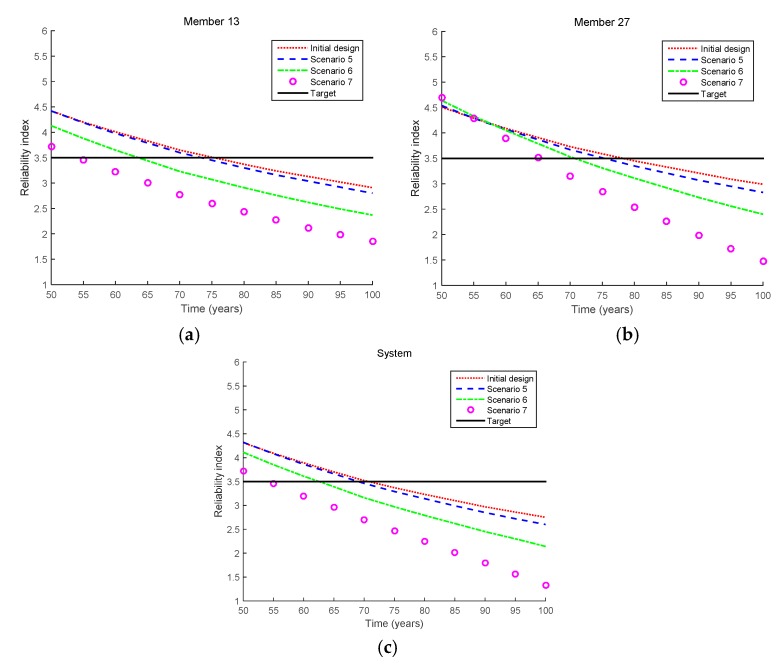
Comparison of updated reliability indices for equality cases (Scenarios 5–7 in Table 3). (**a**) Member 13; (**b**) Member 27; (**c**) System.

Figure 9 shows the reliability index results for the equality cases, and the updated indices in Scenarios 5–7 are shown in Figure 10. From Scenarios 5 to 7, the measured crack size increases from 0.1 to 1.0 mm. It is clearly seen that the longer the crack size measured, the more likely the members and the system are to fail. In addition, the fatigue lives of Member 13, Member 27, and the bridge system in Scenarios 5–7 are shown in Table 5.

#### 4.2.3. Mixed Cases

**Figure 11:**
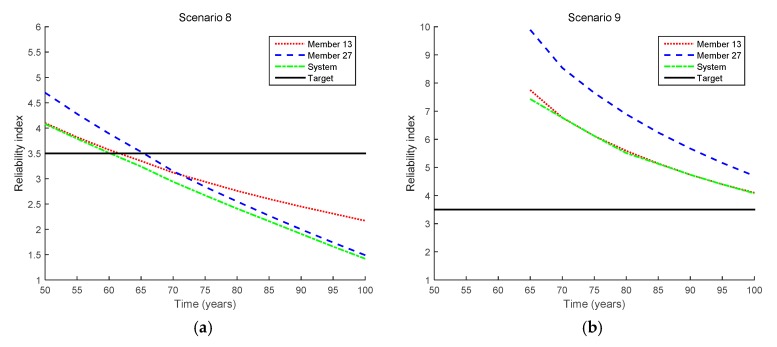
Reliability updating results by the proposed method for inequality cases (Scenarios 8 and 9 in Table 3). (**a**) Scenario 8; (**b**) Scenario 9.

**Figure 12:**
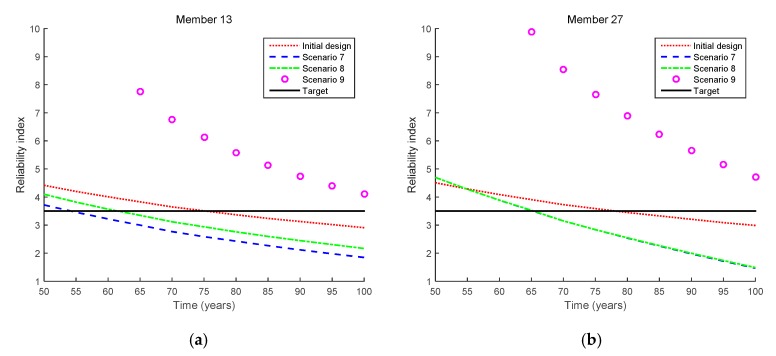

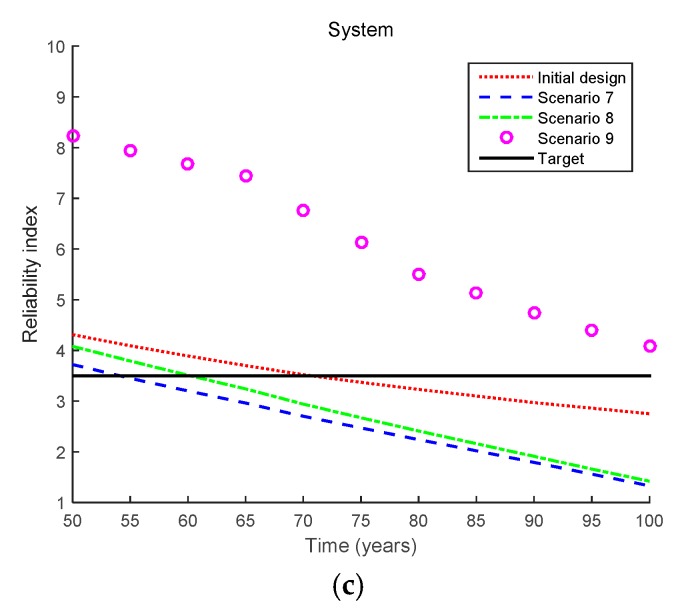
Comparison of updated reliability indices for equality cases (Scenarios 8 and 9 in Table 3). (**a**) Member 13; (**b**) Member 27; (**c**) System.

Figure 11 shows the results obtained by the proposed method for the mixed cases (i.e., Scenarios 8 and 9), and Figure 12 shows how the reliability indices are updated in these scenarios. For comparison purposes, the updated reliability indices in Scenario 7 are also presented in the figure. First, it is clearly seen that the updated failure probability becomes considerably lower in Scenario 8 than in Scenario 7 even though the same crack size is observed at the same inspection time point. This is due to the additional inequality events (i.e., no more cracks being detected at the other locations) observed in the mixed case for Scenario 8. However, after Members 13 and 27 are repaired, the updated reliability indices are increased. Furthermore, the fatigue lives of Member 13, Member 27, and the bridge system are shown in Table 6.

Lastly, it is noteworthy that the reliability updating could be performed in only a few minutes in each scenario (using a general personal computer with 3.60 GHz CPU and 8.00 GB RAM), which demonstrates the computational efficiency of the proposed method. MCS is not a feasible option because it would require a huge number of samples to obtain converged results. As discussed by Lee and Song [38], it would require 3 × 10^5^ samples and a similar number of FE analyses to achieve 5% c.o.v. in the probability result, which is practically impossible even though each analysis takes only a few minutes. However, the proposed method makes system reliability updating feasible and provides considerable savings in terms of computational time.

## 5. Conclusions

This study presents a new system reliability approach to evaluate the fatigue failure risk of railway bridges and update their prior risk information in various scenarios of inspection and repair. The proposed method can handle a varying-amplitude load and can update the system-level risk of fatigue failure for truss bridges after inspection and repair. To demonstrate the proposed method, it is applied to a numerical example of an actual railway bridge. The analysis results show that the proposed method facilitates the updating of reliability estimates for railway bridges subjected to the risk of fatigue-induced failures. In addition, the effects of various inspection and repair scenarios on the reliability updating are investigated in the numerical example, such as the number of inspections, crack-detecting resolution, inspection interval, inspection location, measured crack length, and repair. The investigation results confirm that the proposed method successfully provides component and system reliability updates under various inspection and repair scenarios.

## Figures and Tables

**Table 1:** 10 members with the maximum stresses.

Member No.	13	27	8	6	7	5	10	9	17	4
Maximum stress (MPa)	32.82	32.48	21.26	20.73	20.73	20.22	20.16	19.26	18.95	18.48

**Table 2:** Statistical properties of random variables.

Random Variable (RV)	Mean	c.o.v.	Distribution Type	Number of RVs
Paris law parameter (*C*)	1.537 × 10^−12^ m/cycle/(MPa·mm)*^m^*	0.2258	Lognormal	10
Initial crack length (*a*^0^)	0.11 mm	1.0	Exponential	10
Initial crack length in repaired member (*a^r^*)	0.11 mm	1.0	Exponential	# of repair events
Detectable crack size (*a^d^*)	1.0 mm	1.0	Exponential	# of inequality events
Crack measurement error (ε*^m^*)	0	0.1 *	Normal	# of equality events
Live load scale factor (*S*)	1	0.1	Lognormal	1

*: standard deviation.

**Table 3:** Hypothetical scenarios of inspection and repair.

Case	Scenario Number	Scenario Description
Inequality	1	No crack is detected in Member 13 (*T_I_* = 50 years & mean of *a^d^* = 1.0 mm)
	2	No crack is detected in Member 13 (*T_I_* = 50 years & mean of *a^d^* = 0.5 mm)
	3	No crack is detected in Member 13 (*T_I_* = 75 years & mean of *a^d^* = 1.0 mm)
	4	No crack is detected anywhere (*T_I_* = 50 years & mean of *a^d^* = 1.0 mm)
Equality	5	0.1 mm crack is found in Member 13 (*T_I_* = 50 years)
	6	0.5 mm crack is found in Member 13 (*T_I_* = 50 years)
	7	1.0 mm crack is found in Member 13 (*T_I_* = 50 years)
Mixed	8	1.0 mm crack is found in Member 13, but nowhere else (*T_I_* = 50 years & mean of *a^d^* = 1.0 mm)
	9	0.5 mm crack is found in Member 13, but nowhere else, and Members 13 and 27 are repaired (*T_I_* = 50 years & mean of *a^d^* = 1.0 mm)

**Table 4:** Fatigue lives for inequality cases (Scenarios 1–4 in Table 3).

Scenario	Fatigue Life (Years)		
	Member 13	Member 27	System
Initial design	75.4	78.2	70.7
Scenario 1	88.8	84.2	79.7
Scenario 2	97.9	88.1	85
Scenario 3	92.5	85.8	81.9
Scenario 4	94.2	88.8	84.2

**Table 5:** Fatigue lives for equality cases (Scenarios 5–7 in Table 3).

Scenario	Fatigue Life (Years)		
	Member 13	Member 27	System
Initial design	75.4	78.2	70.7
Scenario 5	73.3	75.3	69
Scenario 6	63.6	70.7	62.5
Scenario 7	54.2	65.3	54.1

**Table 6:** Fatigue lives for mixed cases (Scenarios 8 and 9 in Table 3).

Scenario	Fatigue Life (Years)		
	Member 13	Member 27	System
Initial design	75.4	78.2	70.7
Scenario 7	54.2	65.3	54.1
Scenario 8	61.6	65.4	60.2
Scenario 9	>100	>100	>100
